# Copy of a Letter from Dr. Eustis, of Boston, to a Friend in London, Respecting the Use of Cold Air and Water, in Fevers

**Published:** 1799-03

**Authors:** W. Eustis

**Affiliations:** Boston


### *Copy of a Letter from Dr.* Eustis, *of Boston, to a Friend in London, respecting the Use of cold Air and Water, in Fevers.*

" Dear Sir,

" YOUR favour of last Spring, accompanied with Dr. Currie's valuable publication on the use to which water may be applied in fevers, was doubly acceptable; as an instance of your friendship and polite attention, and as gratifying my professional taste in a very favourite point. From an experience acquired by some years practice in the army, I had learned, that exposure to cold and weather was not an evil of such frightful magnitude to persons in fevers, as is generally imagined by the inhabitants of cities, and especially those in higher life: on the contrary, of febrile patients that were lodged in barns, sheds, and other open places, a greater number recovered than of those who were thought to be better accommodated in the close rooms of dwelling-houses, and regular hospitals: and whether sick or wounded, if I could procure the area of a Dutch barn, they were considered as well lodged, and the patients very generally recovered: this must be understood in reference to the summer and the beginning of autumn. Neither the dews of night, nor any accidental wetting from showers, were deemed half so dangerous, as the poisonous air constantly generated by the sick, respired and re-respired until the patient in a close room faulters and sinks, while under the very eye of his physician, seldom adverting to the real cause.

" From the strong ideas necessarily imprinted on my mind by this experience, and observing many successful instances of exposing to currents of air in symptoms of the small-pox, together with an early conviction that many sick die of their own atmosphere; I always have been, and am an enthusiast for *air*.

" The application of water, and that cold, I found recommended by Dr. Jackson, of Jamaica, who had used it in the fevers of that climate with considerable success; and after reading Dr. Currie's book before mentioned; I was determined to give it a fair trial.

" When the epidemic or yellow fever broke out here last summer, many were affected with a heat of body, so intense as to render painful the hand of a healthy subject when long in contact: this appears to me to have been the genuine *causus* of the antients. In one of these cases, a boy of twelve years of age, subject to delirium, occasional stupor, picking the bed-cloaths, and and other ſymptoms commonly reputed fatal, I reſolved to make trial of the cold bath. In the morning I took him from his bed placed in a wood-ſhed in the yard, (to avoid the heat of the chamber,) ſtripped him naked, had him led to the pump, (ſea-water not being at hand,) and poured over his head and body a common pail of pump-water. On returning to his bed he found himſelf cooled and refreſhed, and the delirium ſenſibly abated. The ſame experiment was made afterwards with ſea-water, and repeated three or four times a day for ſeveral days ſucceſſively, with the ſame good effect. Once or twice the patient, when hot and uneaſy, inſtinctively aſked for the bath; and once, when he was particularly hot, I omitted the uſual precaution of wiping the water from his body, and returned him to his mattraſs wet, as conceiving that the water evaporated too quickly. His fever began to abate, and after ſome days, of a debilitated ſtate, during which his recovery appeared doubtful, he gradually acquired ſtrength, and now enjoys perfect health.

" In another inſtance of an athletic ſubject who, after bleeding and other evacuations, complained of an inexplicable ſort of pain in the head and ſmall of the back; relief and a cure were manifeſtly the reſult of ſponging the parts occaſionally with ſea-water, and expoſing the patient to ſtrong currents of air. In ſeveral inſtances the cold bath has been attended with the moſt beneficial effects; as I learn from other phyſicians; altho' in ſome it has failed. One patient was preſerved by ſponging the whole body with vinegar rendered cold by cakes of ice, where this was preferred to the cold bath; the effect however is preciſely ſimilar. In my own practice, this remedy has certainly ſaved two, if not three patients, who would have fallen under the ordinary treatment; and it has alſo proved ſerviceable in many other caſes. One fact, and that of no ſmall importance, ſeems fairly eſtabliſhed; and in this I agree with Dr. JACKSON, that no injury can reſult from the application of cold water: at leaſt I never knew of ſuch an inſtance. Is not the idea of *catching cold* in a *fever*, according to the uſual acceptation of the phraſe, an abſurdity in terms? Cold is preciſely the thing wanted in fevers; there is ſafety therefore and no danger in taking it.

" I admit that, while our epidemic prevailed, many caſes occurred, in which it would have been improper to uſe the cold bath. In judicious hands however, it is a moſt powerful remedy: in no others ſhould *any* remedy be placed. I have met with one or two inſtances where the heat of the whole body was ſo intenſe, that nothing but the mechanical action of air and water on the ſurface could abate it; let me add, however, that in commending the free and fearleſs uſe of water, I ſpeak of our climate in

 the the ſummer ſeaſon. In the winter I have uſed the partial bath with evident good effect."

Vale! Salve!

